# Immunotherapeutic Potential of Oncolytic H-1 Parvovirus: Hints of Glioblastoma Microenvironment Conversion towards Immunogenicity

**DOI:** 10.3390/v9120382

**Published:** 2017-12-15

**Authors:** Assia L. Angelova, Milena Barf, Karsten Geletneky, Andreas Unterberg, Jean Rommelaere

**Affiliations:** 1 Department of Tumor Virology (F010), German Cancer Research Center (DKFZ), 69120 Heidelberg, Germany; m.barf@dkfz-heidelberg.de (M.B.); j.rommelaere@dkfz-heidelberg.de (J.R.); 2 Department of Neurosurgery, University Hospital, 69120 Heidelberg, Germany; karsten.geletneky@mail.klinikum-darmstadt.de (K.G.); andreas.unterberg@med.uni-heidelberg.de (A.U.)

**Keywords:** oncolytic virotherapy, oncolytic H-1 parvovirus, recurrent glioblastoma, tumor microenvironment, immune suppression, immunogenic conversion, oncolytic immunotherapy

## Abstract

Glioblastoma, one of the most aggressive primary brain tumors, is characterized by highly immunosuppressive microenvironment. This contributes to glioblastoma resistance to standard treatment modalities and allows tumor growth and recurrence. Several immune-targeted approaches have been recently developed and are currently under preclinical and clinical investigation. Oncolytic viruses, including the autonomous protoparvovirus H-1 (H-1PV), show great promise as novel immunotherapeutic tools. In a first phase I/IIa clinical trial (ParvOryx01), H-1PV was safe and well tolerated when locally or systemically administered to recurrent glioblastoma patients. The virus was able to cross the blood–brain (tumor) barrier after intravenous infusion. Importantly, H-1PV treatment of glioblastoma patients was associated with immunogenic changes in the tumor microenvironment. Tumor infiltration with activated cytotoxic T cells, induction of cathepsin B and inducible nitric oxide (NO) synthase (iNOS) expression in tumor-associated microglia/macrophages (TAM), and accumulation of activated TAM in cluster of differentiation (CD) 40 ligand (CD40L)-positive glioblastoma regions was detected. These are the first-in-human observations of H-1PV capacity to switch the immunosuppressed tumor microenvironment towards immunogenicity. Based on this pilot study, we present a tentative model of H-1PV-mediated modulation of glioblastoma microenvironment and propose a combinatorial therapeutic approach taking advantage of H-1PV-induced microglia/macrophage activation for further (pre)clinical testing.

## 1. Glioblastoma: Current Clinical State-Of-The-Art

Glioblastoma is the most common and aggressive primary brain tumor. It has a dismal prognosis and is typically characterized by largely inevitable recurrence within six months to one year after initial treatment [1,2]. The current standard of care for newly diagnosed patients includes maximal surgical resection and subsequent concurrent treatment with radiation and temozolomide (TMZ), followed by adjuvant chemotherapy [3]. Due to glioblastoma invasiveness and frequent location in injury-prone brain areas controlling essential motor and cognitive functions, radical resection is only feasible in a subset of patients [1]. Furthermore, at recurrence only a minority of the patients is eligible for second surgery [4], which is so far the only approach firmly associated with improved survival if complete resection of the recurrent tumor is accomplished [5]. In recurrent glioblastoma patients, standard cytotoxic agents, corticosteroids, TMZ rechallenge, carboplatin and irinotecan are applied, among other agents, to palliate symptoms and improve quality of life, but fail to prolong the time to progression [6]. In 2009, the humanized monoclonal antibody bevacizumab targeting tumor angiogenesis (through the vascular endothelial growth factor (VEGF)), was approved for the treatment of recurrent glioblastoma patients on the basis of its ability to achieve superior progression-free survival (PFS), yet in the lamentable absence of meaningful overall survival (OS) improvement [7].

## 2. Immune Suppression in Glioblastoma Patients

Glioblastoma patients generally show profound local and systemic immune suppression [8]. This tumor-supportive environment is characterized by reduced cluster of differentiation (CD)4^+^ T lymphocyte counts, increased Forkhead-Box-Protein P3 (FoxP3^+^) regulatory T cell (Treg) numbers, and elevated circulating myeloid-derived suppressor cells [9,10,11]. Defective receptor expression by monocytes and impaired dendritic cell (DC) function has also been described [12]. Glioblastoma cells and associated microglia/macrophages express various factors that contribute to the establishment of functional immune suppression in the tumor microenvironment. Immunosuppressive and tumor-promoting cytokines, such as interleukin (IL)-1, IL-6, IL-10, transforming growth factor (TGF)-β, colony stimulating factor (CSF) 1, VEGF, and prostaglandin E2 (PGE2), among others, are secreted and impair anti-glioma immune responses. Furthermore, glioblastoma and associated microglia/macrophage cells upregulate several surface molecules (CD95, CD70, programmed cell death ligand 1 (PD-L1)) which inhibit T cell activation and induce T cell apoptosis, thus reducing the numbers of tumor-infiltrating immune effector cells [8,13]. In consequence, the profound immune masking results in tumor evasion from host immune surveillance and leads to subsequent tumor growth and invasion [13]. These observations, together with unavoidable recurrence and resistance of recurrent glioblastoma to conventional treatments, prompted the search for new, immune-targeted therapeutic options to reverse tumor-/tumor microenvironment-driven immune suppression and activate antitumor immune responses.

## 3. Immune-Targeted Therapeutic Approaches for Glioblastoma

Data from preclinical studies provided convincing evidence that immunotherapy is able to promote efficient anti-glioma immune responses [14,15]. Modulation of human cytomegalovirus (CMV)-specific DCs, application of glioma stem cell-antigens-loaded DC vaccines, cytotoxic T lymphocyte-associated protein 4 (CTLA-4) and PD-1 blockage, etc., are among the numerous extensive works (as reviewed in detail in [14,15]) that have been recently done and allowed for a clinical translation of glioblastoma immune targeting. Several immunotherapy approaches are currently under clinical evaluation, including tumor vaccination, immune checkpoint inhibition and adoptive T cell transfer.

### 3.1. Tumor Vaccination

In newly diagnosed glioblastoma patients, Rindopepimut—an epidermal growth factor receptor variant III (EGFR vIII)-specific peptide vaccine—reached phase III clinical testing but disappointingly, when combined with TMZ, failed to add significant OS benefit compared to TMZ alone [16]. This was ascribed to the relatively low percentage of newly diagnosed glioblastomas which express this EGFR mutant [17]. Indeed, the vast heterogeneity of tumor-specific antigen expression in glioblastoma is a major factor limiting the tumor vaccination approach. Hope in that regard has been raised by the identification of ten novel glioblastoma-associated tumor antigens [18] whose curative potential as a multi-peptide vaccine, using CD8^+^ T cell epitopes, has been tested in phase I/II clinical trials [14]. A phase III clinical assessment of a DC-based vaccine is currently active and results are awaited.

### 3.2. Immune Checkpoint Inhibition

The first Food and Drug Administration (FDA)-approved immune checkpoint inhibitor was the humanized anti-CTLA-4 antibody ipilimumab. Although an improved OS was achieved in a phase III melanoma clinical trial [19], ipilimumab showed severe immune-related adverse effects [20] and a clinical utility limited to only a small subset of glioblastoma patients [14]. Other immune checkpoint inhibitors are presently under clinical evaluation in glioblastoma patients, in particular anti-PD-1 (nivolumab, pembrolizumab) and anti-PD-L1 (MEDI4736) antibodies.

### 3.3. Adoptive T Cell Transfer

One clinical trial, in which autologous T cells specific for glioblastoma-expressed CMV antigens were adoptively transferred to recurrent glioblastoma patients, reported significant OS prolongation [21]. Another trial of autologous therapy with CMV-specific T cells is ongoing. Also ongoing are clinical studies using adoptive transfer of autologous T cells with chimeric antigen receptor (CAR) targeting different glioblastoma-associated antigens [14,15].

Despite the impressive research progress recently made in cancer/glioblastoma immunotherapy, it has to be noted that some of the approaches were associated with immune-related adverse effects, imposing the need for concerned risk–benefit assessment. Some studies have reported gastrointestinal, dermatological and endocrine toxicities attributable to immune checkpoint blockade [22]. Furthermore, life-threatening neurotoxicity and other severe complications, including on-target off-tumor toxicities, were documented after CAR T cell infusion [23].

## 4. Need for Novel Immunotherapeutic Strategies: Oncolytic Viruses

As apparent from above, an improved arsenal of immunotherapeutic agents is still needed. Recently, cancer immunotherapy using oncolytic viruses (OVs) has gained much attention and raised considerable hopes [24,25,26,27]. These agents possess the ability of infecting and killing malignant cells without causing harm to normal tissues. This oncoselectivity is a complex phenomenon and is largely due to the dependence of OV life cycle on various tumor cell-specific factors. OV-induced tumor cell toxicity coupled with host immune system stimulation warrant OV clinical development as targeted multimodal cancer therapeutics.

## 5. Oncolytic H-1 Parvovirus

An emerging candidate is the rodent H-1 protoparvovirus (H-1PV), the smallest among all OVs. H-1PV is endowed with natural anticancer activity and is nonpathogenic for humans. These properties, together with the lack of pre-existing immunity in the human population [28], the potential of H-1PV for application via multiple routes (intratumoral, intravenous, intranasal), and its capacity to cross the blood–brain barrier, make this virus a suitable tool for oncolytic virotherapy of several tumor entities, including those of the central nervous system [29,30,31,32]. In addition, H-1PV oncolysis-associated proinflammatory host immune responses observed in preclinical studies raise hopes that parvovirotherapy may offer a safe alternative to current immune-targeted approaches [33].

## 6. Immunotherapeutic Potential of Oncolytic H-1PV: Preclinical Evidence

### 6.1. In Tumor Models Other than Glioma

The ability of H-1PV-infected tumor cells to activate antitumor immune responses has been demonstrated in several preclinical cancer models, in particular hepatoma [34], melanoma [35], pancreatic [36], colorectal [37,38] and nasopharyngeal [39] carcinomas. In these models, H-1PV-infected tumor cell lysates induced DC maturation, proinflammatory cytokine secretion, tumor-associated antigen cross-presentation, and T/Natural Killer (NK) cell stimulation.

### 6.2. In Glioma Models

In contrast to pancreatic cancer and melanoma, for which extensive preclinical work demonstrated enhanced immunogenicity of H-1PV-infected versus non-infected tumors, considerably less is known about H-1PV-infected glioma interactions with the host immune system. In glioma-bearing immunocompetent animals, spleno- and lymphadenomegaly are observed upon infection with minute virus of mice (an autonomous protoparvovirus closely related to H-1PV), together with increased interferon-gamma (IFN-γ) production in spleen and tumor-draining lymph nodes. Proinflammatory cytokine release and upregulated CD80/83/86 expression were detected in antigen-presenting cells, including microglia [40]. In regard to H-1PV, the only preclinical evidence gathered so far comes from an orthotopic rat glioma model, in which H-1PV treatment was able to eradicate even advanced tumors [41]. This therapeutic effect was however present only if the host immune system was intact: T cell depletion impaired H-1PV-induced tumor regression. Importantly, the sole presence of T cells, in the absence of H-1PV treatment, was not sufficient to cause glioma suppression. These observations argue for a role of host T cell responses in H-1PV-promoted oncosuppression [32].

## 7. Immunotherapeutic Potential of H-1PV in Glioblastoma Patients: First Evidence of Immunogenic Tumor Microenvironment Conversion

Several OVs have been tested in glioma clinical trials and hold considerable promise as novel targeted brain tumor therapeutics. The most advanced are the clinical studies using herpes simplex virus (HSV), Newcastle disease virus (NDV), as well as adeno-, reo-, vaccinia, measles, polio-, and vesicular stomatitis virus (VSV) [42,43]. ParvOryx01 (NCT01301430) was the pilot clinical trial of an oncolytic parvovirus, H-1PV, in patients with recurrent glioblastoma [44]. This trial investigated the application of escalating parvovirus doses via either intravenous or intracerebral routes. In addition to assessing H-1PV safety, tolerability, pharmacokinetics, shedding and maximum tolerated dose, tumor tissue samples were acquired at resection and allowed the analysis of glioblastoma cells and glioblastoma microenvironment nine days after parvovirotherapy. The results of the ParvOryx01 clinical trial have been recently published [45]. Together with the promising clinical observations (reliable safety, good tolerability, well predictable pharmacokinetics, induction of neutralizing antibodies in a virus dose-dependent manner while providing a window for booster H-1PV reapplications, and extended median survival in comparison with recent meta-analyses), several intriguing findings arose from the in-situ analyses of resected parvovirus-treated tumors.

### 7.1. Glioblastoma Infiltration with Immune Cells

In comparison with non-parvovirus-treated (historical) controls, increased tumor infiltration with CD45^+^/CD3^+^/CD8^+^ T lymphocytes was observed in some ParvOryx01 patients. Tumor-infiltrating CD4^+^ and CD25^+^ cells were also present, but importantly, FoxP3^+^ Treg cells were only a minor subfraction of the tumor immune cell infiltrate [45]. Of note, tumor-infiltrating cytotoxic T cells (CTLs) were found to be PD-1-negative.

### 7.2. Activation Status of Glioblastoma-Infiltrating Immune Cells

The activated state of tumor-infiltrating CTLs was demonstrated by the abundant expression of granzyme B and the presence of perforin [45]. The CD4^+^ helper T cell activation marker CD 40 ligand (CD40L) was also expressed in parvovirus-treated glioblastomas. Surprisingly, CD40L expression was also detected in non-lymphocyte-infiltrated glioblastomas, hinting at CD40L cellular source other than CD4^+^ T cells.

### 7.3. CD40L Expression by Glioblastoma Cells

**Figure 1:**
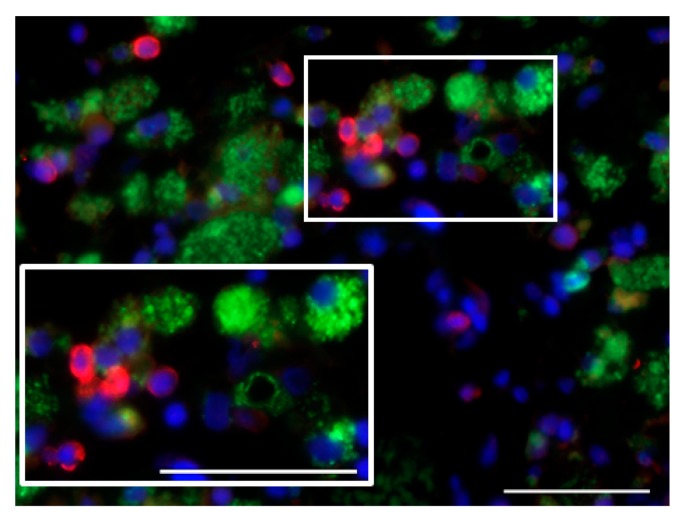
Intra- and perivascularly located cluster of differentiation (CD)40^+^ cells lacking CD68 expression infiltrate the tumor of an intravenously parvovirus-treated glioblastoma patient. CD40, red; CD68, green; DAPI, blue. Scale bars: 50 μm.

In line with the above, CD40L expression was detected in non-macrophage, EGFR-positive, i.e., most likely glioblastoma cells. In contrast, CD40 expression was not observed, either on tumor or on tumor-associated microglia/macrophage (TAM) cells. Interestingly, CD40L expression in glioblastoma cells was first reported in 2015 and found to be a positive prognostic factor, while co-expression of both CD40 and CD40L in glioblastoma cells correlated with negative prognosis [46]. It is noteworthy that in the ParvOryx01 study, CD40^+^ cells with a non-macrophage phenotype were observed in the tumor blood vessel lumen in patients who received H-1PV by intravenous infusion (Figure 1). With the limitation imposed by the unavailability of tumor samples from time points later than nine days after treatment, we hypothesize that CD40-expressing peripheral blood DCs or monocytes may be recruited to H-1PV-infected tumors and interact with CD40L^+^ glioblastoma cells.

### 7.4. Proinflammatory Cytokine Production in Glioblastoma Microenvironment

Two proinflammatory cytokines, IFN-γ and IL-2, were detected in the glioblastoma microenvironment of ParvOryx01 patients who also showed increased tumor infiltration with lymphocytes [45]. IL-12 was not found at detectable levels, at least at the single time point at which tissue material was collected. With this limitation imposed by the trial’s risk-minimizing design, the kinetics of proinflammatory cytokine production in parvovirus-treated glioblastomas could not be followed, and a transient, short lasting expression may be missed. The cellular source of IFN-γ and IL-2 was not identified within the frame of the trial, but two types of cells (tumor-infiltrating CD4^+^ T cells and/or activated TAM, see below) appear as the most likely candidates.

### 7.5. Induction of Cathepsin B Expression

**Figure 2:**
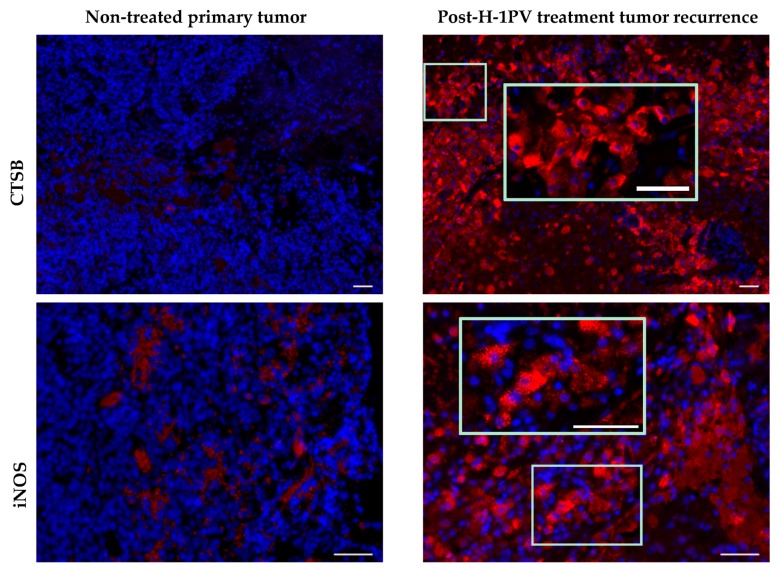
Comparative analysis of cathepsin B (CTSB) and inducible nitric oxide (NO) synthase (iNOS) expression in non-H-1 parvovirus (H-1PV)-treated primary tumor and in the second tumor recurrence (two months after H-1PV treatment of the first recurrence). CTSB, red; iNOS, red; DAPI, blue. Scale bars: 50 μm.

**Figure 3:**
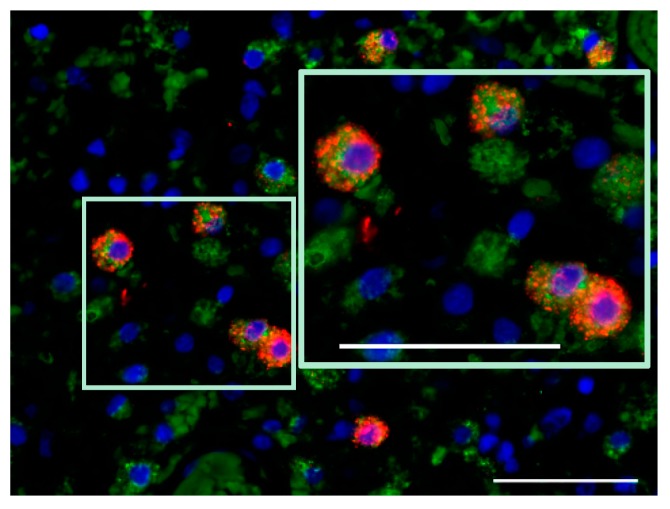
Microglia/macrophage (CD68^+^) phenotype of iNOS-expressing cells in the second tumor recurrence (two months after H-1PV treatment of the first recurrence). CD68, green; iNOS, red; DAPI, blue. Scale bars: 50 μm.

In agreement with preclinical data demonstrating cathepsin B induction and cytosolic translocation in H-1PV-infected human glioma cells [47], the expression of this lysosomal protease was significantly increased in ParvOryx01 patients, when compared with historical recurrent glioblastoma cases. Contrary to initial expectations, cathepsin B expression was observed not only in tumor cells, but mostly in TAM [45]. As increased cathepsin B production correlates with microglial activation [48], and induction of glioma apoptosis by microglia-derived cathepsin B has been demonstrated in vitro [49], first steps were undertaken within the frame of the ParvOryx01 study, to assess glioblastoma-associated microglia/macrophage activation state. Expression of inducible nitric oxide (NO) synthase (iNOS), a marker of classical (proinflammatory, macrophage phenotype M1) microglia/macrophage activation, was assessed in four ParvOryx01 patients, selected on the basis of strong CD68 positivity. While in one patient iNOS expression was not revealed, iNOS-positive cells were detected in the other three patients’ tumors at day nine after H-1PV application. The availability of resection material from the primary tumor and a second recurrence (respectively preceding and following first recurrence subjected to H-1PV treatment) provided the unique opportunity to analyze glioblastoma microenvironment in a non-parvovirus-exposed tumor, as well as at a later time point after virotherapy. Compared with the control primary glioblastoma, the first recurrence (resected at day nine post-treatment) showed a clear induction of both cathepsin B [45] and iNOS expression in TAM cells, which persisted and even increased in the second recurrence at two months after virus application (Figure 2 and Figure 3). These data should be put together with the in vitro demonstration of human macrophage activation as a result of their abortive infection with H-1PV [36], and with the constitutive NO and oxygen species production in human promonocytic cell clones surviving H-1PV-induced apoptosis [50]. It should also be stated that in a recent study of iNOS expression in grade IV glioblastoma patients, the iNOS levels detected failed to positively correlate with survival [51]. Altogether, the above data provide first hints on H-1PV ability to trigger M1 microglia/macrophage polarization in the glioblastoma microenvironment. This concept is still speculative but would be worth corroborating through further (pre)clinical studies, as it is of major relevance for future combinatorial approaches using H-1PV and other immunotherapeutic agents.

## 8. Other Oncolytic Viruses and Tumor Microenvironment Modulation

In a recent editorial, de Vries et al. emphasized that the most successful OV-based strategy will be the one taking advantage of these viruses’ capacity for modifying the tumor microenvironment [52]. Indeed, several OVs were reported to exert positive effects in regard to tumor microenvironment modulation.

### 8.1. Preclinical Studies

Zamarin et al. observed marked infiltration of distant tumors with effector, but not Treg cells, in melanoma-bearing mice treated with Newcastle disease virus [53]. Intratumoral administration of an oncolytic adenovirus decreased tumor-infiltrating Treg cell numbers and stimulated IFN-γ-producing CD8^+^ T cells in a mouse glioblastoma model [54]. Recently, the immunotherapeutic potential of a recombinant polio/rhinovirus chimera was demonstrated in vitro in high-grade glioblastoma [55]. Other preclinical studies using bladder, colon, and breast cancer animal models also pointed to recombinant poxviruses’ positive effects on the immunological components of the tumor microenvironment, as summarized in [52].

### 8.2. Clinical Studies

In a clinical setting, positive effects (dense tumor infiltration with effector T cells, decreased Treg and myeloid-derived suppressor cell numbers, and increased IFN-γ production by CD4^+^ and CD8^+^ tumor-infiltrating lymphocytes) of recombinant vaccinia and HSV, and of measles virus on the tumor microenvironment were demonstrated in melanoma and cutaneous T cell lymphoma patients [56,57,58,59]. Remarkably, responses were observed in both injected and non-injected melanoma lesions, in patients who were intratumorally treated with a granulocyte-macrophage colony-stimulating factor-encoding second-generation HSV [60]. The added value of poxvirus-mediated intratumoral expression of specific tumor-associated antigens was supported by clinical trials in patients with prostate and locally advanced pancreatic cancer [52]. However, clinical data on antitumor immune responses and tumor microenvironment modulation in OV-treated patients remain presently limited [61].

## 9. Tumor Microenvironment Modulation by H-1PV: Current Hypothesis and Perspective

Glioblastoma microenvironment is marked by profound immunosuppression [8]. This tumor-supportive microenvironment [51,62] is established by glioblastoma stem cells, tumor and TAM cells. The pilot ParvOryx01 study gathered initial evidence of tumor microenvironment immunogenic conversion in recurrent glioblastoma patients who underwent local or systemic H-1 parvovirotherapy (Table 1).

**Figure 4:**
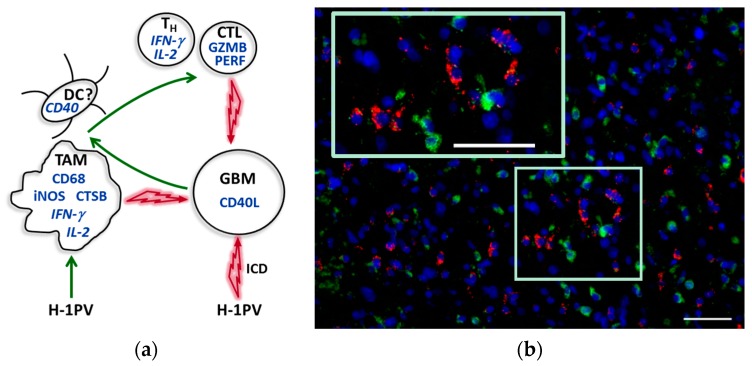
Central role of TAM in the immunogenic conversion of glioblastoma microenvironment upon H-1PV infection. (**a**) Tentative model based on existing (pre)clinical data. Interacting cells are presented with corresponding proinflammatory products (in italics if cellular source unproved). The identification of CD40^+^ myeloid cells as being denditric cells (DCs) is putative. Green and red arrows indicate activating and cytotoxic interactions, respectively. For details, see main text. T_H_, helper T cell; CTL, cytotoxic T lymphocyte; GZMB, granzyme B; PERF, perforin; GBM, glioblastoma; ICD, immunogenic cell death; (**b**) Activated TAM (CD68, green) in CD40L-positive (red) tumor region. Scale bars: 50 μm.

Based on both preclinical experience and the observations from ParvOryx01 patients, we believe that H-1PV treatment may lead to glioblastoma-associated microglia/macrophage activation. This is supported by the increased expression of CD68, cathepsin B and iNOS in tumors of H-1PV-treated patients, in comparison with historical non-parvovirus-treated controls. As a direct result of their abortive parvovirus infection and/or indirectly, via proinflammatory mediators released by infected tumor cells, microglia/macrophages get activated and may exert toxic effects on neighboring glioblastoma cells through cathepsin B and NO release (Figure 4). Another likely scenario, supported by evidence from animal models [40], is that microglia/macrophage capacity for expressing immune costimulatory molecules (usually strongly compromised in glioblastoma) may also be increased upon H-1PV-triggered activation. Tumor tissue collection in this first trial, primarily designed to assess safety, did not allow performing such studies, but in future H-1PV glioblastoma trials, tumor infiltration with DCs, the CD40/CD40L axis, and costimulatory molecule expression on TAM and DCs will be worth investigating.

In conclusion, H-1PV—an oncolytic parvovirus which showed excellent safety and tolerability upon local and systemic application in glioblastoma patients—may represent a novel tool for therapeutic TAM activation (Figure 4). Immunogenic conversion of TAM is the subject of intensive investigations, as exemplified by the recent preclinical demonstration of the antifungal agent’s amphotericin B capacity for restoring the suppressed ability of glioblastoma-associated microglia/macrophages to target brain tumor-initiating cells [65]. We suggest that in future attempts at prolonging time to glioblastoma recurrence, H-1PV (alone or with other treatments targeting TAM) may be combined with other immunotherapeutic modalities, in particular CAR T cell-based strategies and immune checkpoint blockade, to achieve synergistic antiglioblastoma effects.

## Figures and Tables

**Table 1:** Main characteristics of glioblastoma microenvironment as described in the literature or observed in H-1 parvovirus-treated glioblastoma patients.

Glioblastoma Microenvironment (Literature Data)	Glioblastoma Microenvironment (First Parvovirus Clinical Trial)
sparse inflammatory infiltrates (except the mesenchymal transcriptional class) [63]	tumor infiltration with CD8^+^/granzyme B^+^ T cells [45]
tumor-infiltrating lymphocyte involvement in inhibitory (e.g., via PD-1) interactions [8]	PD-1-negative tumor-infiltrating T cells
increased numbers of tumor-infiltrating Treg cells [64]	Treg cells only scarcely detected [45]
M2 tumor-supportive tumor-associated microglia/macrophage (TAM) phenotype [51]	detection of markers of M1 TAM polarization (CD68, CTSB, iNOS) [45]
release of immunosuppressive factors and anti-inflammatory cytokines [8]	detection of proinflammatory cytokines (IFN-γ, IL-2) [45]

PD-1, programmed cell death protein 1; Treg, regulatory T cell; M2, macrophage phenotype 2; CD, cluster of differentiation; CTSB, cathepsin B; iNOS, inducible nitric oxide synthase; IFN-γ, interferon gamma; IL-2, interleukin 2.
